# Supplementary material for: Whole genome landscapes of uveal melanoma show an ultraviolet radiation signature in iris tumours
Source: Nat Commun. 2020 May 15;11:2408. doi: 10.1038/s41467-020-16276-8 (PMC7229209; doi:10.1038/s41467-020-16276-8)
Supplement: Supplementary file 1 — Supplementary Information [file 41467_2020_16276_MOESM1_ESM.pdf]

SUPPLEMENTARY INFORMATION

Whole genome landscapes of uveal melanoma show an ultraviolet radiation signature in iris tumours, Johansson *et al.*

Supplementary Figure 1. Evolutionary conversation of mutated residues.

Supplementary Figure 2. Survival analysis of *TP53* or *RPL5.*

Supplementary Table 1. Significantly Mutated Genes

a

Amino acid residue (Human)

Species	9	10	11	12	13	**14**	15	16	17	18	19
Homo sapiens (Human)	V	C	V	R	V	**R**	P	L	N	S	R
Pan troglodytes (Chimpanzee)	V	C	V	R	V	**R**	P	L	N	S	R
Tursiops truncatus (Bottlenose Dolphin)	V	C	V	R	V	**R**	P	L	N	S	R
Bos taurus (Cow)	V	C	V	R	V	**R**	P	L	N	H	R
Canis familiaris (Dog)	V	C	V	R	V	**R**	P	L	N	D	R
Mustela putorius furo (Ferret)	V	C	V	R	V	**R**	P	L	N	N	R
Dasypus novemcinctus (Armadillo)	V	C	V	R	V	**R**	P	L	N	T	R
Loxodonta africana (Elephant)	V	C	V	R	V	**R**	P	L	N	S	R
Oryctolagus cuniculus (Rabbit)	V	C	V	R	V	**R**	P	L	N	S	R
Mus musculus (House Mouse)	V	C	V	R	V	**R**	P	L	N	S	R
Xenopus tropicalis (Frog)	V	C	V	R	V	**R**	P	L	I	Q	R
Danio rerio (Zebrafish)	V	C	V	R	V	**R**	P	L	I	K	R

Amino acid residue (Human)

Species	1033	1034	1035	1036	1037	**1038**	1039	1040	1041	1042	1043
Homo sapiens (Human)	V	K	D	N	E	**I**	I	E	Q	Q	R
Pan troglodytes (Chimpanzee)	V	K	D	N	E	**I**	I	K	Q	Q	R
Tursiops truncatus (Bottlenose Dolphin)	V	E	D	N	E	**L**	I	E	Q	Q	R
Bos taurus (Cow)	A	E	D	N	E	**L**	T	E	Q	Q	R
Canis familiaris (Dog)	V	E	D	N	E	**L**	T	E	Q	Q	R
Mustela putorius furo (Ferret)	V	E	D	N	E	**L**	T	E	Q	Q	R
Dasypus novemcinctus (Armadillo)	V	G	D	N	E	**L**	I	E	Q	Q	R
Loxodonta africana (Elephant)	I	G	D	N	E	**L**	S	E	Q	E	R
Oryctolagus cuniculus (Rabbit)	V	T	D	D	E	**A**	S	E	Q	Q	R
Mus musculus (House Mouse)	V	K	L	A	A	**T**	L	E	E	C	Q
Xenopus tropicalis (Frog)	E	G	E	S	E	**E**	L	Q	S	L	R
Danio rerio (Zebrafish)	-	-	-	-	-	**-**	-	-	-	-	-

Amino acid residue (Human)

Species	246	247	248	249	250	**251**	252	253	254	255	256
Homo sapiens (Human)	G	A	A	G	V	**R**	L	K	E	G	C
Pan troglodytes (Chimpanzee)	G	A	A	G	V	**R**	L	K	E	G	C
Tursiops truncatus (Bottlenose Dolphin)	G	V	E	G	L	**R**	L	K	E	G	C
Bos taurus (Cow)	G	A	E	G	L	**R**	L	K	E	G	C
Canis familiaris (Dog)	G	A	E	G	V	**R**	L	K	E	G	C
Mustela putorius furo (Ferret)	G	A	E	G	V	**R**	L	K	E	G	C
Dasypus novemcinctus (Armadillo)	G	A	E	G	V	**R**	L	K	E	G	C
Loxodonta africana (Elephant)	G	A	E	G	V	**R**	L	K	E	G	C
Oryctolagus cuniculus (Rabbit)	G	A	E	G	V	**R**	L	K	E	G	C
Mus musculus (House Mouse)	D	T	S	G	V	**L**	L	-	-	-	-
Xenopus tropicalis (Frog)	G	A	E	G	A	**R**	F	K	E	G	C
Danio rerio (Zebrafish)	G	S	E	G	V	**R**	L	K	E	G	C

Amino acid residue (Human)

Species	1816	1817	1818	1819	1820	**1821**	1822	1823	1824	1825	1826
Homo sapiens (Human)	A	K	L	Q	E	**K**	I	Q	E	L	K
Pan troglodytes (Chimpanzee)	A	K	L	Q	E	**K**	I	Q	E	L	K
Tursiops truncatus (Bottlenose Dolphin)	A	K	L	Q	E	**K**	V	Q	E	L	K
Bos taurus (Cow)	A	K	L	Q	E	**K**	V	Q	E	L	K
Canis familiaris (Dog)	A	K	L	Q	E	**K**	I	Q	Q	L	K
Mustela putorius furo (Ferret)	A	K	L	Q	E	**K**	I	Q	Q	L	K
Dasypus novemcinctus (Armadillo)	A	K	L	E	E	**K**	I	Q	E	L	K
Loxodonta africana (Elephant)	A	E	L	Q	K	**K**	I	Q	E	L	K
Oryctolagus cuniculus (Rabbit)	A	K	L	Q	E	**K**	I	Q	E	L	K
Mus musculus (House Mouse)	L	D	T	E	H	**L**	R	Q	T	L	K
Xenopus tropicalis (Frog)	A	V	T	V	E	**R**	D	Q	L	M	E
Danio rerio (Zebrafish)	C	D	T	E	E	**L**	K	E	E	L	K

Amino acid residue (Human)

Species	1093	1094	1095	1096	1097	**1098**	1099	1100	1101	1102	1103
Homo sapiens (Human)	E	L	K	K	Q	**Q**	E	I	V	A	Q
Pan troglodytes (Chimpanzee)	E	L	K	K	Q	**Q**	Q	I	V	A	Q
Tursiops truncatus (Bottlenose Dolphin)	E	L	K	K	Q	**Q**	E	I	V	A	Q
Bos taurus (Cow)	E	L	K	K	Q	**Q**	D	I	V	A	Q
Canis familiaris (Dog)	E	L	K	K	Q	**Q**	E	I	L	V	Q
Mustela putorius furo (Ferret)	E	L	K	R	Q	**Q**	E	I	I	T	Q
Dasypus novemcinctus (Armadillo)	E	L	K	K	Q	**Q**	E	I	I	T	R
Loxodonta africana (Elephant)	E	L	K	K	Q	**Q**	E	I	M	A	Q
Oryctolagus cuniculus (Rabbit)	D	L	K	K	Q	**Q**	E	I	V	V	Q
Mus musculus (House Mouse)	E	Y	H	K	L	**I**	S	L	A	K	E
Xenopus tropicalis (Frog)	K	V	K	E	L	**Q**	G	E	I	N	R
Danio rerio (Zebrafish)	-	L	K	E	Q	**I**	F	S	L	T	Q

b

Amino acid residue (Human)

	686	687	688	689	690	**691**	692	693	694	695	696
Homo sapiens (Human)	I	I	E	D	S	**R**	E	A	T	H	S
Pan troglodytes (Chimpanzee)	I	I	E	D	S	**R**	E	A	T	H	S
Tursiops truncatus (Bottlenose Dolphin)	I	I	E	D	S	**G**	E	A	T	H	S
Bos taurus (Cow)	I	I	E	D	S	**R**	E	A	T	H	S
Dasypus novemcinctus (Armadillo)	I	I	E	D	S	**R**	E	A	T	H	S
Canis familiaris (Dog)	I	I	E	D	S	**R**	E	A	T	H	S
Loxodonta africana (Elephant)	I	I	E	D	S	**H**	E	A	T	H	S
Oryctolagus cuniculus (Rabbit)	I	I	E	D	S	**R**	E	A	T	H	S
Dasypus novemcinctus (Armadillo)	I	I	E	D	S	**H**	E	A	T	H	S
Mus musculus (House Mouse)	I	I	E	E	S	**R**	E	A	T	H	S
Xenopus tropicalis (Frog)	I	L	E	A	S	**Q**	E	D	T	R	T

c

Amino acid residue (Human)

	298	299	300	301	302	**303**	304	305	306	307	308
Homo sapiens (Human)	P	M	G	A	Q	**D**	L	I	S	K	L
Papio anubis (Baboon)	P	T	G	A	Q	**D**	L	I	S	K	L
Bos taurus (Cow)	P	L	G	A	Q	**D**	L	I	Y	K	L
Canis familiaris (Dog)	P	T	G	A	Q	**D**	L	I	S	K	L
Mustela putorius furo (Ferret)	P	S	G	A	Q	**D**	L	I	S	K	L
Loxodonta africana (Elephant)	P	T	G	A	Q	**D**	L	I	S	K	L
Dasypus novemcinctus (Armadillo)	P	P	G	A	Q	**D**	L	I	S	K	L
Oryctolagus cuniculus (Rabbit)	P	A	G	A	Q	**D**	L	I	S	K	L
Mus musculus (House Mouse)	P	S	G	A	Q	**D**	L	I	S	K	L
Danio rerio (Zebrafish)	S	E	G	A	R	**D**	L	I	S	K	L
Xenopus tropicalis (Frog)	S	D	G	S	K	**D**	L	I	S	K	L

d

Amino acid residue (Human)

	1475	1476	1477	1478	1479	**1480**	1481	1482	1483	1484	1485
Homo sapiens (Human)	S	L	S	S	S	**C**	V	P	D	S	S
Pan troglodytes (Chimpanzee)	S	L	S	S	S	**C**	V	P	D	S	S
Dasypus novemcinctus (Armadillo)	S	V	S	F	S	**G**	V	T	N	S	T
Mustela putorius furo (Ferret)	S	L	S	F	S	**C**	V	T	D	S	P
Tursiops truncatus (Bottlenose Dolphin)	S	L	S	L	S	**G**	V	T	D	S	P
Bos taurus (Cow)	S	L	S	F	S	**C**	V	T	D	S	P
Oryctolagus cuniculus (Rabbit)	-	-	-	-	S	**L**	I	C	N	S	P
Mus musculus (House Mouse)	P	L	S	F	-	**C**	G	A	D	S	P
Danio rerio (Zebrafish)	E	E	Y	K	D	**F**	A	L	Q	E	-

Supplementary Figure 1. Evolutionary conversation of mutated residues. a, CENPE **b,** BUB1B **c,** AURKB or **d,** CENPF. Altered residue outlined in red. Conservation data were obtained from the Aminode online tool (http://www.aminode.org) described in Chang *et al.* (Science Reports 2018, doi:10.1038/s41598-018-19744-w)

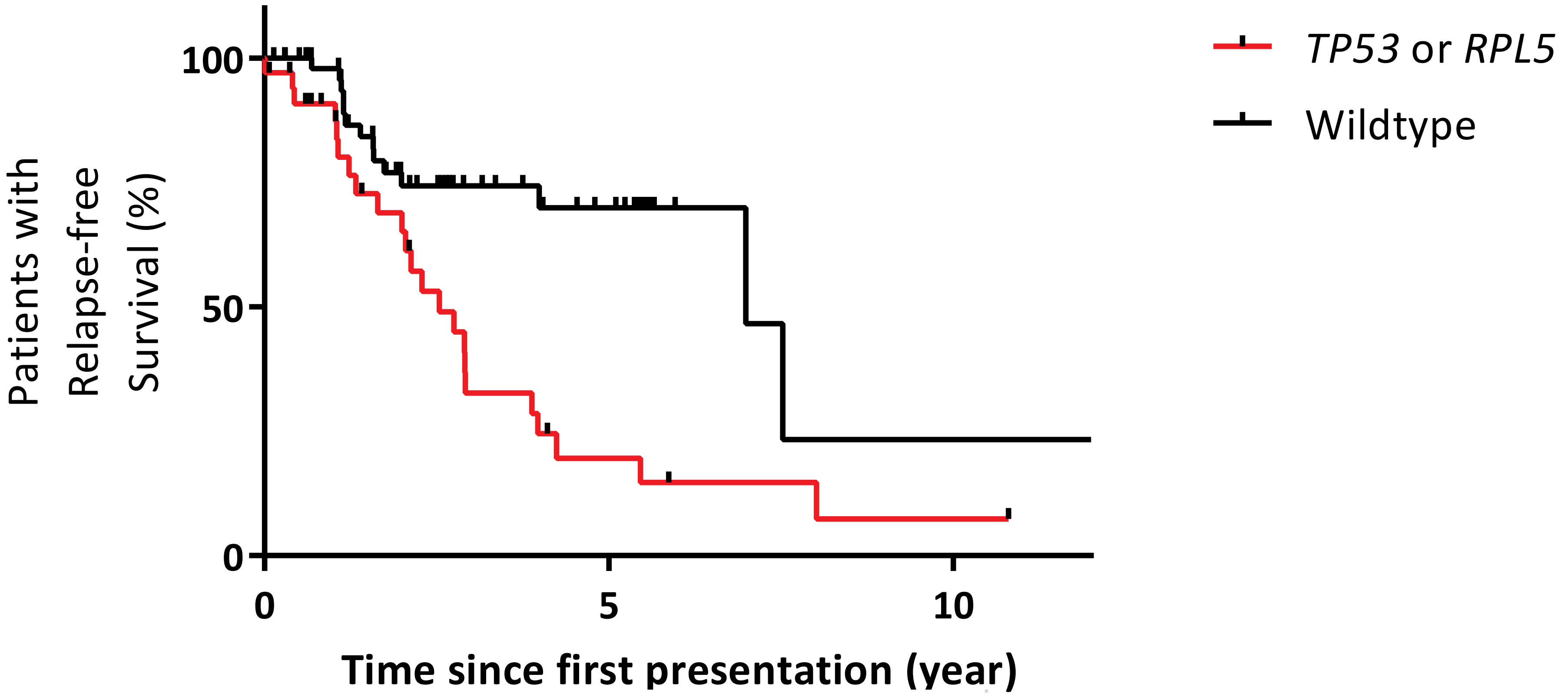

No. at Risk

**Wildtype**	53	45	28	20	16	13	3	2	1	1	1
TP53* or *RPL5	34	26	17	8	6	4	2	2	2	1	1

Supplementary Figure 2. Kaplan-Meier estimate of relapse-free survival for UM patients with (red) or without (blue) alterations in *TP53* or *RPL5.*

Supplementary Table 1 | Significantly Mutated Genes

SYMBOL	MutSigCV q-value	Oncodrive-fm q-value	OncodrivClust q-value
GNAQ	0	0	6.7E-08
EIF1AX	1.0E-11	2.9E-12	0.04
BAP1	0	0	ns
GNA11	ns	0	6.7E-08
PLCB4	ns	0.008	6.7E-08
SF3B1	ns	0	3.6E-07
TP53	ns	5.5E-05	ns
RPL5	ns	0.002	ns
CENPE	ns	0.022	ns
