# Supplementary material for: Whole genome landscapes of uveal melanoma show an ultraviolet radiation signature in iris tumours
Source: Nat Commun. 2020 May 15;11:2408. doi: 10.1038/s41467-020-16276-8 (PMC7229209; doi:10.1038/s41467-020-16276-8)
Supplement: Supplementary file 3 — Description of Additional Supplementary Files [file 41467_2020_16276_MOESM3_ESM.pdf]

Description of Additional Supplementary Files

File Name: Supplementary Data 1

Description: Clinical and Mutation Data

File Name: Supplementary Data 2

Description: Coding Mutations (substitutions and indels)
