# Supplementary material for: Whole genome landscapes of uveal melanoma show an ultraviolet radiation signature in iris tumours
Source: Nat Commun. 2020 May 15;11:2408. doi: 10.1038/s41467-020-16276-8 (PMC7229209; doi:10.1038/s41467-020-16276-8)
Supplement: Supplementary file 6 — Reporting Summary [file 41467_2020_16276_MOESM6_ESM.pdf]

nature research

Corresponding author(s): Nicholas Hayward

Last updated by author(s): Mar 26, 2020

Reporting Summary

Nature Research wishes to improve the reproducibility of the work that we publish. This form provides structure for consistency and transparency in reporting. For further information on Nature Research policies, see Authors & Referees and the Editorial Policy Checklist.

Statistics

For all statistical analyses, confirm that the following items are present in the figure legend, table legend, main text, or Methods section.

n/a	Confirmed	
☐	☒	The exact sample size (n) for each experimental group/condition, given as a discrete number and unit of measurement
☒	☐	A statement on whether measurements were taken from distinct samples or whether the same sample was measured repeatedly
☐	☒	The statistical test(s) used AND whether they are one- or two-sided<br>*Only common tests should be described solely by name; describe more complex techniques in the Methods section.*
☒	☐	A description of all covariates tested
☒	☐	A description of any assumptions or corrections, such as tests of normality and adjustment for multiple comparisons
☒	☐	A full description of the statistical parameters including central tendency (e.g. means) or other basic estimates (e.g. regression coefficient) AND variation (e.g. standard deviation) or associated estimates of uncertainty (e.g. confidence intervals)
☐	☒	For null hypothesis testing, the test statistic (e.g. F, t, r) with confidence intervals, effect sizes, degrees of freedom and P value noted<br>*Give P values as exact values whenever suitable.*
☒	☐	For Bayesian analysis, information on the choice of priors and Markov chain Monte Carlo settings
☒	☐	For hierarchical and complex designs, identification of the appropriate level for tests and full reporting of outcomes
☒	☐	Estimates of effect sizes (e.g. Cohen's d, Pearson's r), indicating how they were calculated

Our web collection on statistics for biologists contains articles on many of the points above.

Software and code

Policy information about availability of computer code

Data collection	Progeny 10
Data analysis	cutadapt 1.9<br>bwa-mem 0.7.12<br>samtools 1.8<br>picard 1.129<br>star 2.5.2<br>mutsigcv 1.3.5<br>oncodrive-fm 1.0.3<br>oncodriveclust 1.0.0<br>fathmm 1.0<br>ascatngs<br>qsv 0.3<br>Ensemble gene model v70<br>COSMIC mutation signature v3

For manuscripts utilizing custom algorithms or software that are central to the research but not yet described in published literature, software must be made available to editors/reviewers. We strongly encourage code deposition in a community repository (e.g. GitHub). See the Nature Research guidelines for submitting code & software for further information.

Data

Policy information about availability of data

All manuscripts must include a data availability statement. This statement should provide the following information, where applicable:

- Accession codes, unique identifiers, or web links for publicly available datasets
- A list of figures that have associated raw data
- A description of any restrictions on data availability

The BAM files have been deposited in the European Genome-phenome Archive (https://www.ebi.ac.uk/ega/) with accession number EGAS00001001552. The source data underlying Fig. 2 are provided as a data source file.

Field-specific reporting

Please select the one below that is the best fit for your research. If you are not sure, read the appropriate sections before making your selection.

☒ Life sciences ☐ Behavioural & social sciences ☐ Ecological, evolutionary & environmental sciences

For a reference copy of the document with all sections, see nature.com/documents/nr-reporting-summary-flat.pdf

Life sciences study design

All studies must disclose on these points even when the disclosure is negative.

Sample size	We recruited as many patients as possible. We considered 103 samples sufficient as it is the largest whole-genome sequencing study of uveal melanoma to date and exceeded the number in the exome sequencing study conducted by TCGA.
Data exclusions	Six samples were excluded because they had estimated tumour cell purity below 30%. Exclusion criteria were not pre-established.
Replication	Each sample was sequenced once.
Randomization	Randomization was not applicable since this study comprised whole-genome sequencing of an unselected series of tumours.
Blinding	Blinding was not applicable as there was no randomization.

Reporting for specific materials, systems and methods

We require information from authors about some types of materials, experimental systems and methods used in many studies. Here, indicate whether each material, system or method listed is relevant to your study. If you are not sure if a list item applies to your research, read the appropriate section before selecting a response.

Materials & experimental systems

n/a	Involved in the study
☒	☐ Antibodies
☒	☐ Eukaryotic cell lines
☒	☐ Palaeontology
☒	☐ Animals and other organisms
☐	☒ Human research participants
☒	☐ Clinical data

Methods

n/a	Involved in the study
☒	☐ ChIP-seq
☒	☐ Flow cytometry
☒	☐ MRI-based neuroimaging

Human research participants

Policy information about studies involving human research participants

Population characteristics	Clinic-based series of UM patients. The mean age of participants was 65 years (standard deviation = 13.6, range: 21 – 91). Fifty-three of 103 (51%) participants were women.
Recruitment	UM patients suitable for surgery and with sufficient fresh-frozen tumour remaining after pathology requirements for whole-genome sequencing were approached. We did not notice any factors that correlated with patients' willingness to donate tissue.
Ethics oversight	All tumour and blood/saliva samples were accrued with written informed patient consent following institutional review board approval from the Human Research Ethics Committees of the QIMR Berghofer Medical Research Institute, the Sydney Local Health District RPAH zone, the University of Western Australia, the Capitol Region of Denmark and the Ludwig Institute for Cancer Research.

Note that full information on the approval of the study protocol must also be provided in the manuscript.
